# Generisch-Net: A Generic Deep Model for Analyzing Human Motion with Wearable Sensors in the Internet of Health Things

**DOI:** 10.3390/s24196167

**Published:** 2024-09-24

**Authors:** Kiran Hamza, Qaiser Riaz, Hamza Ali Imran, Mehdi Hussain, Björn Krüger

**Affiliations:** 1 Department of Computing, School of Electrical Engineering and Computer Science, National University of Sciences and Technology (NUST), Islamabad 44000, Pakistanhimran.mscs18seecs@seecs.edu.pk (H.A.I.);; 2 Department for Epileptology, University Hospital Bonn, 53127 Bonn, Germany; bkrueger@uni-bonn.de

**Keywords:** generic deep model, IMU, MEMS, wearable sensors, smart wearable

## Abstract

The Internet of Health Things (IoHT) is a broader version of the Internet of Things. The main goal is to intervene autonomously from geographically diverse regions and provide low-cost preventative or active healthcare treatments. Smart wearable IMUs for human motion analysis have proven to provide valuable insights into a person’s psychological state, activities of daily living, identification/re-identification through gait signatures, etc. The existing literature, however, focuses on specificity i.e., problem-specific deep models. This work presents a generic BiGRU-CNN deep model that can predict the emotional state of a person, classify the activities of daily living, and re-identify a person in a closed-loop scenario. For training and validation, we have employed publicly available and closed-access datasets. The data were collected with wearable inertial measurement units mounted non-invasively on the bodies of the subjects. Our findings demonstrate that the generic model achieves an impressive accuracy of 96.97% in classifying activities of daily living. Additionally, it re-identifies individuals in closed-loop scenarios with an accuracy of 93.71% and estimates emotional states with an accuracy of 78.20%. This study represents a significant effort towards developing a versatile deep-learning model for human motion analysis using wearable IMUs, demonstrating promising results across multiple applications.

## 1. Introduction

The IoHT is a more comprehensive form of the Internet of Things. The IoHT platform’s major purpose is to autonomously intervene from geographically diverse locations by providing low-cost preventative or active healthcare treatments. IoHT communication, integration, computation, and interoperability are powered by several low-power embedded systems with limited computing capabilities. Because it may be utilized in a wide range of medical professions, the Internet of Health Things (IoHT) technology is important among physicians and patients in today’s healthcare environment. It consists of tiny groups of medical equipment sensors and actuators that can gather, analyze, and exchange data over the internet [1]. The applications of Inertial measurement units have recently experienced a significant boom due to the growing significance of the Internet of Things (IoT) [2,3,4]. The consumer market is currently flooded with a wide range of digital gadgets equipped with IMUs, including but not limited to smartwatches, smartphones, smart fitness bands, smart insoles, etc. IMU sensors are low-cost, are easy to wear on the subject’s body, and can collect low-level kinematics at higher frequencies for a longer duration in unconstrained environments. The analysis of such low-level gait signatures’ data can provide valuable insights into human motion analysis such as the prediction of the affected state of a person, the re-identification of a person in a closed-loop scenario, the estimation of the health and well-being of a person through the prediction of activities of daily living, etc.

The remarkable progress in micro-electromechanical systems (MEMSs) has led to substantial advancements across various scientific disciplines. Inertial sensors, like accelerometers and gyroscopes, are critical MEMS components integral to IMUs. Due to their favorable attributes, such as affordability, low energy consumption, lightweight design, and portability, inertial sensors are extensively utilized in numerous industries. Data gathered from these sensors can be analyzed for sophisticated motion analysis using deep learning methodologies. In particular, inertial sensors are employed in multiple applications, including HAR [5,6,7], HER [8,9], person identification [10], sports activity recognition [11,12], risk assessment for musculoskeletal disorders [13], and so on.

Over the years, there has been an increase in the use of IMU-based deep learning models due to their cost-effectiveness and accurate analysis capabilities. However, many of the current deep learning approaches are highly specialized, are designed for specific application areas, and often lack generalizability. Their performance tends to degrade significantly with even slight changes in low-level kinematics, making them unsuitable for adaptation to new, similar problems without substantial re-engineering. This work aims to develop a generalized model capable of performing robust and realistic gait analysis and inference across a broader spectrum of scenarios. In this paper, we offer a generic deep model called Generisch-Net, which can be trained and validated across three different application areas: (1) affect state prediction, (2) Re-ID (Re-ID), and (3) HAR. The results demonstrate that the model tends to achieve acceptably high accuracies for all three application areas. The key contributions are listed below.

- We introduce Generisch-Net, a novel generic BiGRUs–convolutional neural network (BiGRU-CNN) designed to analyze human motion using wearable IMUs, such as those found in smartwatches and smartphones. This model has been trained for human activity recognition (HAR), human emotion recognition (HER), and Re-ID (Re-ID) tasks (see Section 4).
- The proposed model has been validated across three datasets, achieving average accuracies of 96.97% for HAR, 93.71% for Person Re-ID, and 78.20% for HER (see Section 5).
- A comparative analysis with existing state-of-the-art application-specific methodologies is provided to justify our approach (see Section 6).

The article is organized as follows: Section 2 offers an in-depth review of recent relevant literature. Section 3 details the datasets used in the study. The comprehensive pipeline of the proposed deep model is outlined in Section 4. Section 5 presents the results and analyses. Lastly, Section 6 provides a conclusive summary of the article.

## 2. Literature Review

Human gait analysis using wearable IMUs has been used for human mobility analysis such as the estimation of human emotions [9], human activities [14,15], the detection of cognitive impairment and falls [16], and even Re-ID. In the context of our work, we concentrate on research that not only shares the same analytical technique but also uses the same datasets. We start with the state-of-the-art literature on HAR, followed by the estimation of emotions and Re-ID.

Zhang et al. [17] created a deep model with an attention mechanism, reporting an F1-score of 94.5% on the WISDM 2011 dataset. Xia et al. [18] designed a model that combines LSTM, CNN, and global average pooling, validated on the WISDM 2011, SMARTPHONE, and OPPORTUNITY datasets, achieving average accuracies of 95.85%, 95.78%, and 92.63%, respectively. Pang et al. [19] presented a model for which an F1-score of 99.13% was reported on the WISDM 11 dataset, featuring two novel modules: the Pyramid Multi-Scale Convolutional Network (PMCN) for detailed receptive field and multiscale representation, and the Cross-Attention Mechanism, which enhances relevant information and suppresses irrelevant data by establishing interrelationships among sensor, temporal, and channel dimensions.

Li et al. [20] proposed a feature-set enhancement mechanism for which input feature maps $F_{\mathrm{in}}$ are first processed through a convolutional neural network (CNN) and subsequently undergo a deconvolution operation. The resultant feature map $F_{\mathrm{enhanced}}$ is then concatenated with $F_{\mathrm{in}}$, yielding an augmented training feature map $F_{\mathrm{train}}=F_{\mathrm{in}}⊕F_{\mathrm{enhanced}}$. This methodology achieved a classification accuracy of 99% on the WISDM 2011 dataset. Nafea et al. [21] introduced a hybrid model combining a CNN and bidirectional long short-term memory (BiLSTM), reporting an average classification accuracy of 98.53% on the same dataset. Ihianle et al. [22] developed a CNN+BiLSTM architecture, reporting a weighted average F1-score of 98% on the WISDM 2019 dataset.

Imran et al. [5] introduced the HARDenseRNN model, combining CNN-BiGRU layers with a direct link between input signals and the BiGRU component. The model was evaluated on the WISDM 2011 and WISDM 2019 datasets. For the WISDM 2011 dataset, it attained an accuracy of 97.29% using 1D input signals derived from the magnitude of 3D acceleration $\sqrt{a_{x}^{2}+a_{y}^{2}+a_{z}^{2}}$ and 98.81% with 3D acceleration components $\left(a_{x},a_{y},a_{z}\right)$. In the 1D context, the precision, recall, and F1-score were all 97%, whereas in the 3D context, these metrics increased to 99%. For the WISDM 2019 dataset, which includes eighteen activities recorded via smartwatches, the model achieved accuracies of 97.5% for 3D acceleration $\left(a_{x},a_{y},a_{z}\right)$ and 98.4% for 6D acceleration and angular velocities $\left(a_{x},a_{y},a_{z},\omega_{x},\omega_{y},\omega_{z}\right)$. In both scenarios, precision, recall, and F1-score were consistently high at 98%.

Wearable inertial sensor-based intelligent systems for identifying human emotions offer several advantages over traditional methods such as facial expression analysis or self-reporting. For instance, wearable sensors’ emotion recognition remains effective even when facial expressions are hidden or not discernible. Wearable sensors equipped with IMUs are particularly suitable for emotion estimation, as they can be attached to the human body non-invasively, can be worn for extended periods, and do not pose any privacy concerns. Zhang et al. [23] achieved an accuracy of 81.2% using smart wristbands with accelerometers to assess neutral, angry, and happy emotions. The study, however, was limited to Chinese respondents and three emotions. Piskioulis et al. [24] used smartphone accelerometers and gyroscopes to identify pleasure and impatience, with accuracy rates of 87.90% and 89.45%, respectively. Reyana et al. [25] used various body sensors to recognize four emotions: happiness, sadness, anger, and neutrality. And they reported accuracies of 80%, 70%, 90%, and 100%, respectively. Quiroz et al. [26], used the data of 50 subjects captured with smart bands’ inertial sensors to identify two emotions, namely joyfulness and sadness. An audio-visual paradigm for the arousal of emotions was employed, and they achieved an accuracy of 75%. Hashmi et al. [27] proposed a set of manually curated features encompassing components derived from the spatiotemporal and wavelet domains. They used a closed-access emotions gait dataset collected with body-mounted smartphone IMUs and recorded six basic emotions, i.e., happiness, sadness, anger, fear, disgust, and surprise. Their findings highlight the potential of leveraging features from these domains to effectively train multiple supervised learning models. An overall classification accuracy of 86.45% was achieved for all six emotions. In another study on the same dataset [9], a CNN-BiGRU model was proposed, and it was able to estimate six emotions with an accuracy of 95%.

Zou et al. [28] developed a deep learning framework for identifying and authenticating individuals in uncontrolled environments using inertial sensor data. They employed smartphones to track human steps in open spaces. In a similar vein, Qiu et al. [29] introduced a model that distinguishes between healthy individuals and those with illnesses. Ahmed et al. [30] utilized visual and inertial sensors to affordably predict human gait. Gohar et al. [10] proposed a BiGRU-based neural network for re-identifying individuals, achieving an accuracy of 86.23% on a wearable IMU dataset.

Researchers have explored and fine-tuned various deep learning models, such as CNNs, LSTMs, and GRUs, for gait analysis and inference in a data and task-specific manner. However, these models often suffer from significant performance degradation with even minor changes in low-level kinematics, raising concerns about their stability and reliability. For example, a model trained on inertial gait data for human activity recognition may experience a sharp decline in performance when applied to different tasks, such as emotion recognition or person re-identification, despite the data type being low-level kinematics. This lack of generalizability limits the applicability of such models and restricts their use in real-world scenarios.

To address this challenge, we introduce Generisch-Net, a generic model architecture designed to operate without domain-specific fine-tuning. Generisch-Net is trained on low-level kinematics data from three distinct datasets, each representing different application areas: human activity recognition, emotion recognition, and person re-identification. The proposed model not only performs well across these tasks but also is lightweight, making it suitable for deployment in low-power IoT edge devices for near real-time decision-making. This broad applicability and this efficiency position Generisch-Net as a practical solution for diverse real-world scenarios.

## 3. Datasets

The proposed generic deep model was trained for three distinct applications: HER, HAR, and Re-ID. For HAR, we utilized two open-access datasets from Fordham University’s WISDM lab, WISDM 2011 [31], and WISDM 2019 [32]. We used a Closed-Access Emotions Dataset [27] for HER. For Re-ID, we used a Closed-Access Re-ID Dataset [10]. All of the datasets are collected with wearable IMUs. In the following sub-sections, brief descriptions of the datasets are given.

### 3.1. Datasets for HAR

For HAR, we relied on two well-known and publicly available datasets from the WISDM lab, namely WISDM 2011 and WISDM 2019. Brief descriptions of both datasets are given below.

#### 3.1.1. WISDM 2011 Dataset

**Figure 1:**
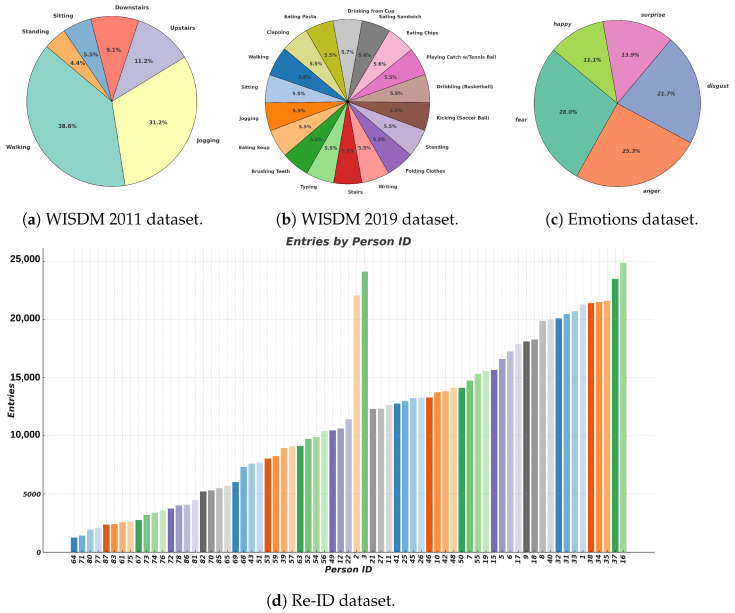
Distribution of classes for different datasets shown here.

The dataset was collected in a controlled environment setting, and it is categorized into six primary classes. The “Walking” class is the most prevalent, containing 424,397 entries and making up 38.6% of the total data. The “Jogging” class follows with 342,176 entries, which constitute 31.15% of the dataset. The “Upstairs” class has 122,869 entries (11.18%), and the “Downstairs” class has 100,427 entries (9.14%). The “Sitting” and “Standing” classes have 59,939 and 48,395 entries, representing 5.45% and 4.40% of the dataset, respectively. During data collection, the phone was placed in the participants’ pockets. The dataset is complete with no missing values, although the class distribution is notably imbalanced, as shown in Figure 1a. The proposed Generisch-Net demonstrates strong performance across all activities, as quantified by precision, recall, and F1-score metrics. Notably, the model achieves these results without employing specific strategies like weighted sampling or adjusting loss functions to address under-represented classes. This suggests that Generisch-Net has an inherent ability to handle class imbalances, evidenced by a high weighted average F1-score of 96% for majority classes and acceptable scores for minority classes. These results highlight the potential of Generisch-Net to manage diverse data distributions, making it a valuable tool for real-world applications where imbalanced datasets are common. Further details are discussed in Section 5.1.2.

#### 3.1.2. WISDM 2019 Dataset

There are eighteen everyday activities in this dataset. 51 volunteers (ages 18–25) provided sensor data from cellphones and smartwatches. For our experiments, we used data collected with a smartwatch. The class distribution of various ADLs is illustrated in Figure 1b, and it is observable that it is adequately balanced. The phone was placed in the pants of the subjects, and the smartwatch was worn on their dominant hands.

### 3.2. Closed-Access Emotions Dataset

This dataset was collected in one of our previous studies [27]. The dataset involved 40 healthy individuals, consisting of 26 men and 14 women, with an average age of 25.1 years. To capture the necessary data, we utilized the internal IMU of a smartphone, which enabled us to calculate 3D accelerations and angular velocities. The smartphones were attached to the participants’ chests using elastic bands. The data collection primarily involved individuals who regularly engage in walking activities. The dataset encompasses six distinct emotions: fear, happiness, anger, sadness, disgust, and surprise. The class distribution is depicted in Figure 1c where the slight class imbalance is observable.

### 3.3. Closed-Access Re-ID Dataset

The individual re-identification dataset was gathered from a prior study of ours [10]. It is demographically diverse, containing data from both Asian and European subjects. A total of 86 volunteer participants (N=86; 49 men and 37 women; age range: 17–72) contributed to the data collection sessions. Data were gathered using two distinct types of sensors: the MPU-6500 IMU embedded in smartphones and the APDM Opal IMU. These sensors were securely attached to the participants’ chests using elastic belts.

The MPU-6500, commonly found in smartphones, includes three sensors: a three-axis gyroscope, a three-axis accelerometer, and, in some cases, a three-axis magnetometer. Its primary function is to measure angular velocity (ω), acceleration (*a*), and, if applicable, magnetic field intensity (*B*). In contrast, the APDM Opal IMU is renowned for its high precision and accuracy in human motion analysis. The Opal IMU, which includes accelerometers, gyroscopes, and magnetometers, is designed specifically for precise tracking of human movement, and it is commonly used in biomechanical research, clinical evaluations, and sports performance analysis. The class distribution of the dataset is illustrated in Figure 1d.

## 4. Methodology

**Figure 2:**
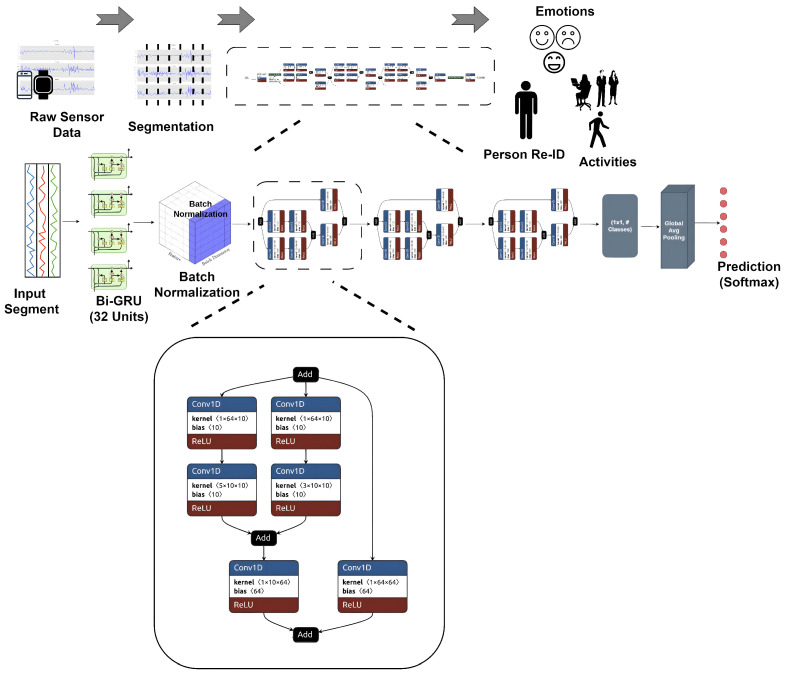
The complete pipeline of the project includes a neural network. IMUs of smartphones and smartwatches are used for data collection. Through segmentation, the long signals are decomposed and passed to Generisch-Net. It has bi-directional GRU layers, followed by three inception-like modules.

This section outlines the methodology used in this study, including preprocessing steps, segmentation processes, and the architecture of the deep learning model. The model consists of BiGRU and CNN layers combined in a special fashion. BiGRUs are used in the Generisch-Net model to account for capturing temporal dependencies existing within sequential data, generated by wearable sensors. Unlike regular recurrent neural networks (RNNs), gated recurrent units (GRUs) can help mitigate the vanishing gradient problem, which is a long-term issue in the training of deep sequences. This is done through a gating mechanism that selectively updates or resets the hidden state, making it easier for the network to keep track of long-term dependencies. A bidirectional approach is further motivated due to the model being able to look both forward and backward in time—a desirable property for interpreting temporal patterns, such as human motion data. Selecting CNN layers, specifically 1D convolutional layers with various kernel sizes (e.g., 1 × 5 and 1 × 3), helps in enhancing the spatial hierarchies of features learned by models. CNN performs local dependencies and automatically captures hierarchal patterns in the context of complex spatial configurations, which is necessary for dealing with complex patterns. It is an interesting design decision to not concatenate feature maps through different kernel sizes and instead add them together. This approach reduces the number of parameters in the model, making it computationally lean and apt for edge devices to perform in real time. Finally, at the end of the model, global average pooling was incorporated to keep the overall complexity of the model as low as possible. The complete workflow of our proposed approach is depicted in Figure 2.

### 4.1. Signal Segmentation

The objective of the study was to come up with a purely deep learning-based solution; therefore, no noise suppression, feature engineering, or any other type of pre-processing was carried out on any of the datasets. Since neural networks require fixed-size input, long-input sequences are segmented into smaller chunks. Here, the segmentation window and stepping size are the important parameters, and they were empirically selected, as shown in Table 1. We conducted experiments to select the best-performing values of segmentation window size and stepping size. Table 1 depicts the comprehensive details of these experiments for each dataset.

### 4.2. Generisch-Net

The study introduces the Generisch-Net model, which stands out from traditional deep learning architectures due to its unique approach of combining feature maps from different-sized kernels through addition, rather than concatenation. This idea significantly reduces the number of model parameters, making it highly suitable for real-time and low-latency applications like Re-ID, HAR, or HER.

To capture both spatial and temporal features effectively, the model incorporates a Bi-directional GRU layer, followed by convolutional layers that operate on the features extracted from the Bi-GRU layer. This combination enhances the model’s performance. Additionally, the model avoids the use of fully connected layers before the softmax layer by employing global average pooling. This design choice not only reduces model complexity but also makes it an ideal option for resource-constrained environments.

The Generisch-Net model utilizes 32 units in its bidirectional GRU (Bi-GRU) layers, as depicted in Figure 2. Following the Bi-GRU layers, there are three convolutional modules referred to as “Diverse-Kernel Modules” (see Figure 2). Batch normalization is applied before the output feature map from the Bi-GRU layer is fed into the CNN modules. Each convolutional module consists of two different-sized convolutional kernels, specifically 1×5 and 1×3, with 10 kernels of each size. Prior to these, 64 1×1 convolutions are applied. The feature maps generated via these kernels are summed, and then 64 1×1 kernels are applied again. The input feature maps are also processed via 64 1×1 kernels, and the resulting feature maps are added to those generated via the 64 1×1 kernels applied after the 1×5 and 1×3 kernels. This setup incorporates residual connections into the module, enabling it to learn identity mappings. Subsequently, global average pooling is applied, followed by a softmax layer.

It is worth noting that the same model was trained for HAR, HER, and Person Re-ID tasks. The tuning focused on parameters such as the segmentation window, step size, epoch, and batch size, adapting them to the specific requirements of each task. The experimental results obtained from multiple benchmark datasets demonstrate the improved performance achieved through this approach.

## 5. Results

Evaluation criteria that provide a more realistic evaluation of the model’s performance and behavior include precision, recall, and F1-score—a combination of the two—in addition to accuracy. This is especially true when working with imbalanced datasets. We used 10 cross-validations for each application and reported the best model. We attained the highest accuracy of 95.624% for WISDM 2011 and 96.98% for WISDM 2019 in the case of HAR. On the closed-access emotion dataset, we obtained 78.2% accuracy for HER. Similarly, using the closed-access Re-ID dataset, an accuracy of 93.71% was obtained. The upcoming subsections provide the specifics.

### 5.1. HAR

We made use of smartphones’ inertial sensor data from WISDM 2011 and smartwatches’ inertial sensor data from the WISDM 19 datasets.

#### 5.1.1. WISDM 2019

**Figure 3:**
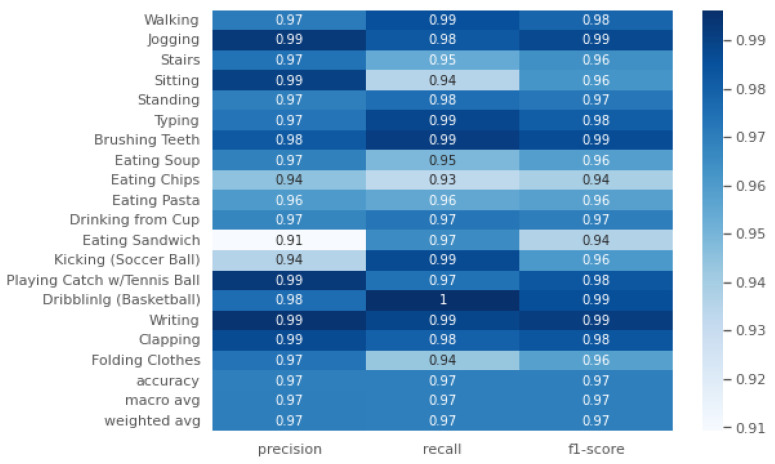
Precision (*P*), recall (*R*) and F1-score ($F_{1}$) for the WISDM 2019 HAR dataset.

**Figure 4:**
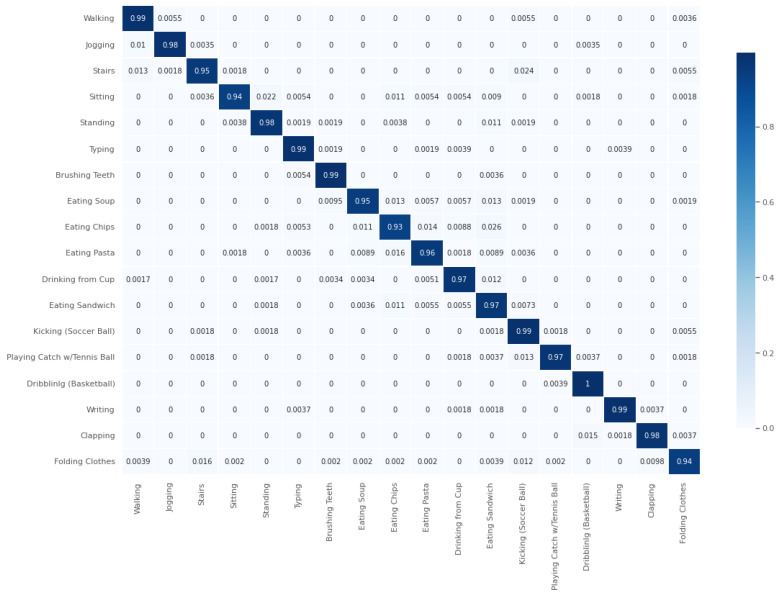
Confusion matrix for WISDM19 for best case of Generisch-Net.

We achieved a test accuracy of 96.978%. The weighted average recall (*R*), F1-score ($F_{1}$), and precision (*P*) were all 97% (Figure 3). From the figure, it can be observed that the class ‘Eating Sandwich’ had the lowest precision at 91% (P=0.91), while its recall was 97% (R=0.97), and its F1-score was 94% ($F_{1}=0.94$). All the other classes had an F1-score ($F_{1}$) above 94%. The confusion matrix in Figure 4 indicates that ‘Eating Sandwich’ was mostly confused with other eating activities, such as ‘Eating Pasta’, ‘Eating Sandwich’, and ‘Eating Chips’, with the highest confusion being 1.1% (1.1%) for ‘Eating Chips’. The highest F1-score ($F_{1}$) of 99% ($F_{1}=0.99$) was achieved for the classes ‘Playing Catch w/Tennis Ball’, ‘Jogging’, ‘Sitting’, and ‘Clapping’ (see Figure 3). The parameters used were as follows: epochs = 70, segmentation size = 256, and step size = 16.

#### 5.1.2. WISDM 2011

**Figure 5:**
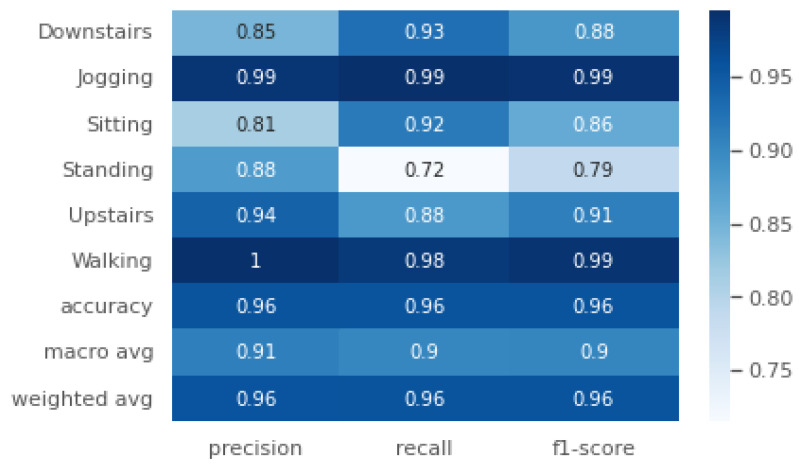
Precision (*P*), recall (*R*) and F1-score ($F_{1}$) for the WISDM 2011 HAR dataset.

**Figure 6:**
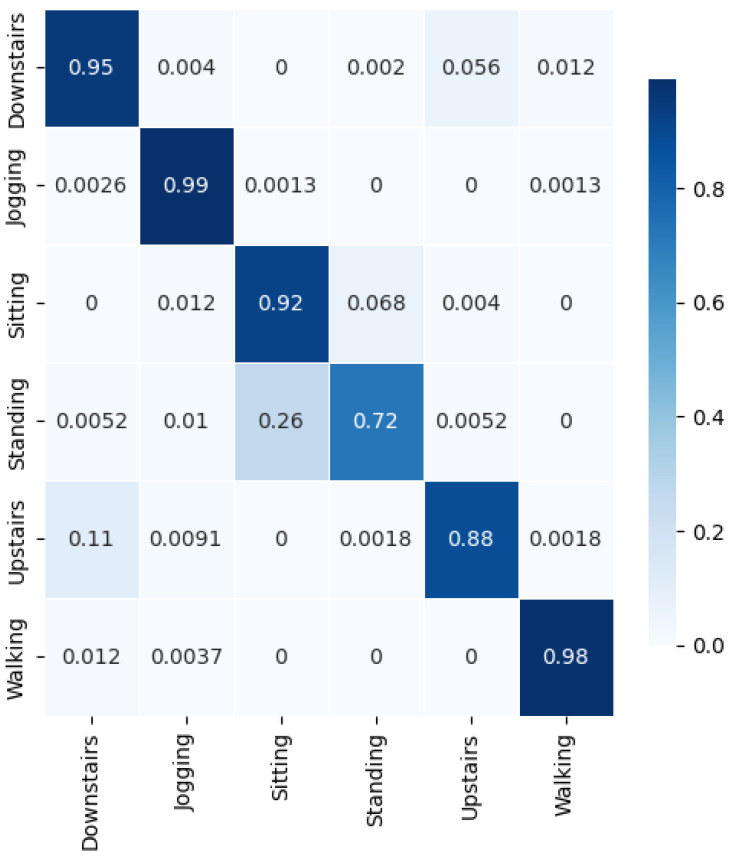
Confusion matrix for WISDM11 for best case of Generisch-Net.

We achieved a test accuracy of 95.624% and a weighted average F1-score ($F_{1}$) of 96%. The recall (*R*), precision (*P*), and F1-score ($F_{1}$) for each class are shown in Figure 5. It can be observed that the minimum F1-score ($F_{1}=0.79$) is for the class ‘Sitting’, which also has the minimum recall (R=0.72). The highest F1-score ($F_{1}=0.99$) was achieved for the classes ‘Walking’ and ‘Jogging’. From the confusion matrix in Figure 6, it can be observed that the ‘Sitting’ class was most confused with ‘Standing’, with a total confusion accuracy of 6.8% (6.8%). Both activities are non-locomotive and involve very little inertial movement, making differentiation challenging. The following tuning parameters were chosen: epochs = 70, segmentation size = 256, and step size = 32.

### 5.2. Re-ID

**Figure 7:**
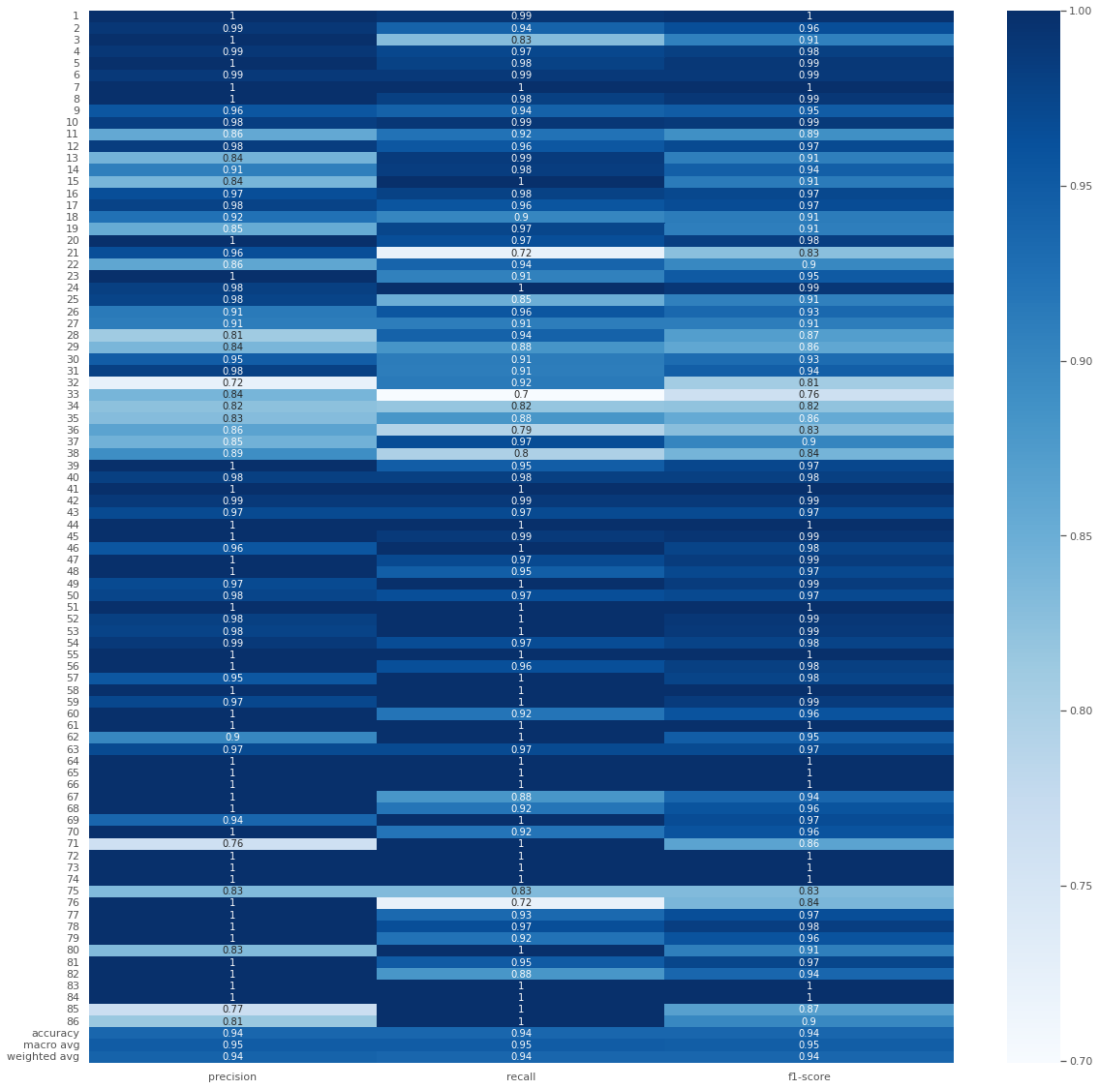
Precision (*P*), recall (*R*) and F1-score ($F_{1}$) for the Re-ID dataset for the best case of Generisch-Net.

**Figure 8:**
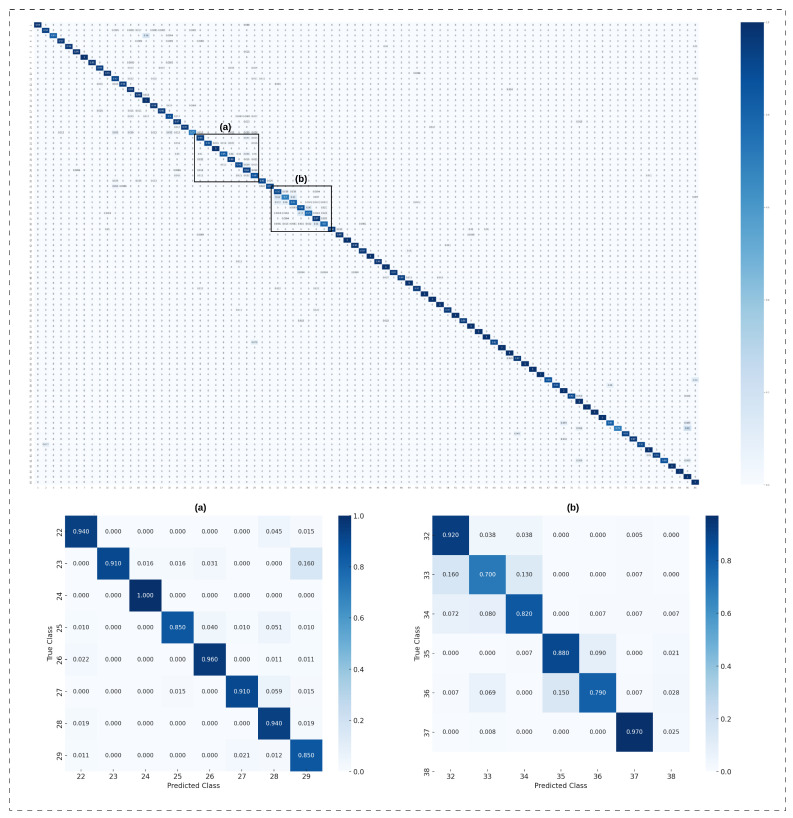
Confusion matrices: the first row shows the confusion matrix for the closed-access Re-ID dataset for the best case of Generisch-Net, and the second row shows zoomed into it for Class 22–29 (**a**) and Class 32–38 (**b**).

The model achieved a test accuracy of 93.713% on the Re-ID dataset, with an average weighted F1-score ($F_{1}$), recall (*R*), and precision (*P*) of 94%. Figure 7 presents the performance report for each individual. Notably, the model outperformed the previous study by Gohar et al. [10]. The minimum F1-score ($F_{1}$), observed for subject ID 31, was 76% ($F_{1}=0.76$), while the highest F1-score ($F_{1}$) of 100% ($F_{1}=1.00$) was achieved for several subjects, including IDs 76, 77, and 82. The confusion matrix in Figure 8 further illustrates the model’s robust performance. The following tuning parameters were employed: epochs = 70, segmentation size = 256, and step size = 32.

### 5.3. HER

**Figure 9:**
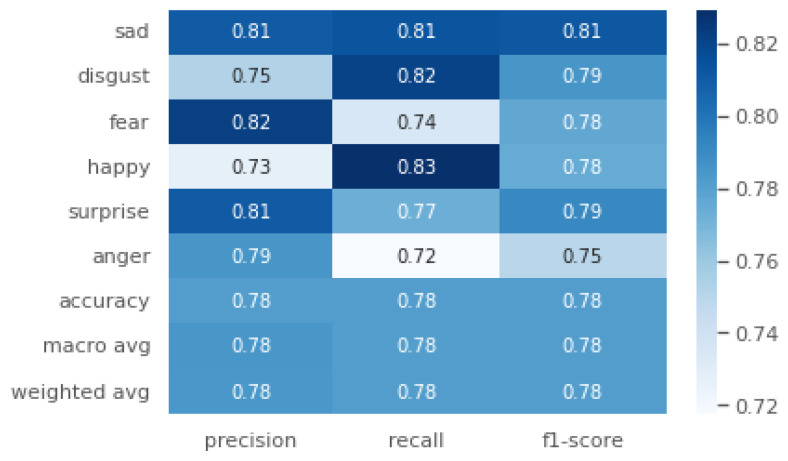
Precision (*P*), recall (*R*) and F1-score ($F_{1}$ for the Closed-Access Emotions Dataset in the best case of Generisch-Net.

**Figure 10:**
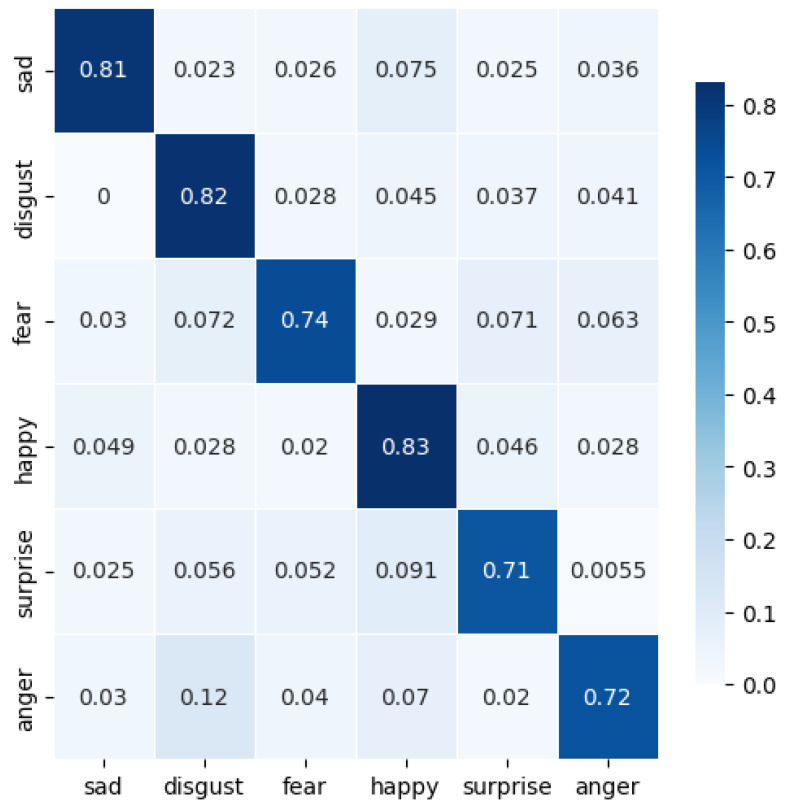
Confusion matrix for closed-access emotions dataset for the best case of Generisch-Net.

Emotion recognition is a relatively challenging problem compared to HAR. We found that the model performed better using only 3D acceleration data, achieving a test accuracy of 78.198% and an average F1-score ($F_{1}$), recall (*R*), and precision (*P*) of 78%. Figure 9 shows the performance report for individual classes. It can be observed that the lowest F1-score ($F_{1}$) was for the ‘anger’ class at 75% ($F_{1}=0.75$), while the highest F1-score ($F_{1}$) was for the ‘sad’ class at 81% ($F_{1}=0.81$). The confusion matrix is shown in Figure 10, indicating that the ‘fear’ class was mostly confused with the ‘happy’ class. The following tuning parameters were chosen: epochs = 70, segmentation size = 256, and step size = 16.

### 5.4. Computational Efficiency

The proposed Generisch-Net is very suitable for deployment in IoT edge devices because of its having low computational and memory overheads, which are critical for efficient real-time IoT edge devices and applications. For the WISDM 2011 dataset, the model comprised 28,616 trainable parameters, occupying only 111.78 KB of memory. Similarly, for the WISDM 2019 dataset, the parameter count slightly increased to 30,272, with a memory size of 118.25 KB. The models for the emotions and Re-ID datasets were identical in size, each containing 29,192 trainable parameters and requiring 114.03 KB of memory. These modest parameter counts and memory footprints enabled Generisch-Net to operate effectively on low-power, low-cost IoT devices, supporting near-instantaneous health monitoring and decision-making. This optimized resource utilization underscores the suitability of Generisch-Net for practical deployment in various Internet of Health Things (IoHT) scenarios, where high performance and efficiency are crucial.

## 6. Conclusions

The aim of the study was to attempt to develop a generic deep neural network for human motion analysis using wearable IMUs. The proposed model is generic, as it was trained and validated on three different applications, i.e., HAR, HER, and Re-ID. The generic model performed fairly well compared to previous studies, as shown in Table 2. For the WISDM 2011 dataset, our presented model achieved an accuracy of 95.624%, outperforming [33]. However, our model was slightly surpassed by the LSTM-CNN model [18], which achieved an accuracy of 95.85%, and the CNN-BiGRU model [9], which achieved 98.81%. Overall, our presented model performed well and showcased competitive accuracy on this dataset.

For the WISDM 2019 dataset, our presented model achieved an accuracy of 96.978%. It outperformed the [32], which achieved an accuracy of 94.4%. The presented model showcased a higher accuracy, indicating superior performance on this dataset. The CNN-BiGRU model presented in [9] outperformed slightly with an accuracy of 98.4%. For Re-ID, our presented model achieved an accuracy of 93.713% and outperformed the [10] BiGRU model, which achieved an accuracy of 86.23%. For the Closed-Access Re-Identification Dataset, our presented model achieved an accuracy of 93.713% and outperformed the [10] BiGRU model, which achieved an accuracy of 86.23%. For the HER, our presented model achieved an accuracy of 78.198%, which was much lower than the CNN-BiGRU model [9] (at 95%). However, a comparison of the results with existing methodologies was not the objective of this study. The development of a generic deep model was our main idea, and our findings show that the proposed model can work on three different application domains; i.e., it is generic. It is important to note that the proposed methodology was validated using 10-fold cross-validation, rather than subject-wise cross-validation. Given the large, diverse, and heterogeneous nature of the dataset in this study, our model demonstrated consistent performance across all folds, indicating its robustness and suggesting that it learned generalized features, rather than overfitting to specific subjects or sessions. We chose 10-fold cross-validation for its balance between computational efficiency and thorough evaluation. Subject-wise cross-validation is computationally expensive, and it can be beneficial in scenarios with limited data or significant variability between subjects.

Generisch-Net’s strong performance stems from key design and architectural choices rooted in deep learning principles and tailored for the precise analysis of wearable sensor data. The model employs a hybrid architecture, combining bidirectional GRUs (BiGRUs) with convolutional neural networks (CNNs) to effectively capture both temporal and spatial information. BiGRU layers excel at learning sequential patterns by capturing temporal dependencies in sensor data, which are crucial for understanding activities, emotions, and identity through low-level kinematics data. The bidirectional nature of GRUs enhances this capability by considering context from both past and future directions, resulting in a more accurate representation of complex movements. Meanwhile, CNN layers specialize in extracting spatial features, with varied kernel sizes enabling the detection of multi-scale features essential for activity and subtle motion recognition. By merging feature maps from different kernels, rather than concatenating them, Generisch-Net maintains a low parameter count, boosting computational efficiency without compromising accuracy—critical for deployment in low-power, memory-constrained devices like wearable sensors and IoT edge devices. Additionally, the use of global average pooling before the final classification layer enhances generalization and reduces the risk of overfitting compared to traditional fully connected layers. This combination of design choices ensures that Generisch-Net not only achieves high accuracy but also operates efficiently on resource-limited edge devices, making it well suited for real-world applications.

## Figures and Tables

**Table 1:** Optimal selection of segmentation window size and step size for each dataset.

Window Size (w)	Step Size (s)	Accuracy (%)
WISDM 11		
128	64	93.54
256	64	94.15
256	32	95.08
WISDM 19		
128	64	83.76
256	64	87.87
256	32	93.65
256	16	96.58
Closed-access Emotions		
128	64	39.63
256	64	38.21
256	32	59.15
256	16	78.63
Closed-access Re-ID		
128	64	77.09
256	64	87.22
256	32	93.12

**Table 2:** Performance comparison with the existing application-specific deep models.

Type & Reference	Accuracy (%)
WISDM 2011 dataset	
[18] LSTM-CNN	95.85
[33] CNN	93.32
[17] CNN with an attention mechanism	96.4
[5] CNN-BiGRU with Direct-link	98.81
Presented model	95.624
WISDM 2019 dataset	
[34] MCBLSTM	96.6 ± 1.47
[32] KNN, DT, RF	94.4
[5] CNN-BiGRU with Direct-link	98.4
Presented Model	96.978
Closed-Access Emotions Dataset	
[27] Traditional ML	86.45
[9] CNN-BiGRU with Raw-link	95
Presented model	78.198
Closed-Access Re-identification Dataset	
[10] BiGRU	86.23
Presented model	93.713

## Data Availability

No new data were created in this study. Data sharing is not applicable to this article.
